# Controversies in the antiphospholipid syndrome: can we ever stop warfarin?

**DOI:** 10.1186/1740-2557-5-6

**Published:** 2008-11-11

**Authors:** Ana G Fonseca, David P D'Cruz

**Affiliations:** 1 The Lupus Research Unit, the Rayne Institute, St. Thomas' Hospital, London, SE1 7EH, UK

## Abstract

Patients with antiphospholipid syndrome are at increased risk for recurrent arterial and venous thrombosis and therefore benefit from long term warfarin therapy. The optimal duration of warfarin therapy after a first venous thromboembolic event is however a matter of some controversy and many questions remain unanswered. After reviewing and analysing the available evidence, we discuss some common scenarios in everyday clinical practice where treatment decisions are difficult.

## Introduction

Patients with antiphospholipid syndrome, an acquired autoimmune thrombophilia, are at risk of both arterial and venous thromboembolic events [1,2]. Indeed, the antiphospholipid syndrome (APS) is defined by vascular thrombosis and/or pregnancy morbidity occurring in the setting of persistently positive antiphospholipid antibodies (aPL) [3]. At least one clinical and one laboratory criteria are required to classify a patient with APS [3]. Relevant aPL laboratory criteria include lupus anticoagulant (LA) and moderate to high titres of anticardiolipin antibodies (aCL), which have tested positive on two occasions at least 12 weeks apart [4]. Recently anti-β2 glycoprotein I antibodies were included in the laboratory classification criteria, although the assays lack standardization and this inclusion has been somewhat controversial [4]. Virtually any vascular territory (venous or arterial) can be affected but deep vein thrombosis of the lower limbs with or without pulmonary emboli is the most common clinical presentation of thrombosis [2].

Antiphospholipid syndrome and antiphospholipid antibodies can occur either alone or in association with systemic connective tissue diseases, most commonly systemic lupus erythematosus (SLE). Approximately half the patients with APS have no underlying systemic autoimmune disease. In SLE, the prevalence of aPL ranges from 12 to 30% for aCL and 15 to 31% for LA, but the prevalence of APS is 10% and this is known to increase with follow – up with an estimated cumulative prevalence of around 30% [1,2,5].

The overall risk of thrombosis is increased in patients with aPL [1]. These antibodies can be identified in 4 to 21% of patients presenting with venous thromboembolism, a significantly higher prevalence than that observed in healthy individuals (1 to 5%) [6-8]. In young patients with stroke, 18% were found to have aPL [9]. LA seems to be more predictive of thrombosis than aCL (odds ratio 11 for LA and 1.6 for aCL, CI 95%) [10]. However, the risk associated with aCL rises when only moderate to high titres are considered [11]. Moreover, in patients with SLE and aPL, the odds ratio for venous thromboembolism was 6.32 when compared with patients without these antibodies [12]. On the other hand, the risk of thrombosis is likely to be low among healthy patients with incidental and transiently positive aPL (< 1% per year) [7]. In patients with aPL, recurrent thromboembolic events are common. A high risk of recurrence has been suggested in retrospective studies, with recurrence rates as high as 69% over 6 years of follow up [13-15].

Thus, patients with APS are considered at high risk of thromboembolic events and warrant effective evidence – based antithrombotic strategies. In patients with both arterial and venous thromboembolic events or more than one thrombotic event there is a consensus that indefinite, life long, anticoagulation therapy is essential to reduce the risk of recurrent thrombotic events [7,16]. However, recurrent arterial events most frequently follow initial arterial events and similarly initial venous events tend to recur as venous events [15]. Following an arterial or venous thrombotic event, secondary prevention with indefinite anticoagulation, initially with low molecular weight heparin or unfractioned heparin, acutely, followed by warfarin is the standard of care. However, defining the adequate length of warfarin therapy remains very controversial [7,16-21]. Given these controversies, recurrent thrombosis in the context of APS and the optimal duration of warfarin treatment for secondary prevention of thrombosis will be discussed. The management of pregnancy loss in APS is beyond the scope of this review.

## From the evidence to the recommendations

Rosove et al and Khamashta et al retrospectively evaluated 70 and 147 patients with APS for a mean of 5 and 6 years from the first thromboembolic event and reported recurrent thromboembolic events (arterial and/or venous) in 53 and 69% of the patients, respectively [13,15]. Finazzi et al and Turiel et al prospectively followed up one cohort of 360 unselected patients with aPL and one cohort of 56 patients with primary APS over 4 and 5 years, respectively [22,23]. Previous thrombosis (arterial or venous) and persistent high titres of anticardiolipin antibodies (IgG > 40 GPL U) were identified as independent predictors of thrombotic events. (Table 1)

**Table 1:** Recurrent thrombosis in the patients with antiphospholipid antibodies

**Study design**	**N**	**Mean age** *Years*	**Entry criteria**	**Patient Characteristics**	**Mean Follow up** *Years*	**Patients with recurrent events** *No. (%)*	**Number of recurrent events** *All (arterial/venous)*	**Reference**
Retrospective	70 48W 22M	45.5 ± 17.3	aPL (aCL/LA) + arterial/venous thrombosis (1^st ^event)	PAPS 51 SLE 14 ITP 5	5.2	37/70 (53%)	54	Rosove MH et al. *Ann Intern Med *1992

Retrospective	19 16W 3M	26 (15–40)	aPL (aCL/LA) + venous thrombosis (1^st ^event)	PAPS 1 SLE 12 Lupus like 6	8	12/19 (63%)	37 (3/34)	Derksen R et al. *Ann Rheum Dis *1993

Retrospective	147 124W 23M	32 (14–66)	aPL (aCL/LA) + arterial/venous thrombosis (1^st ^event)	PAPS 62 SLE 66 Lupus like 19	7	101/147 (69%)	186 (75/111)	Khamashta M et al. *N Eng J Med *1995

Prospective	360 242W 118M	39 (2–78)	aPL (aCL/LA) (117 aPL pt with previous arterial/venous thrombosis)	SLE 69 Lupus like 66	4	25/117 (21.3%)	25	Finazzi G et al. *Am J Med *1996

Prospective	412 181W 231M	60.2	Venous thrombosis (1^st ^event) allocated 6 months warfarin	-	4	20/68 aCL+ (29%) 47/344 aCL- (14%)	67 (-/67)	Schulman S et al. *Am J Med *1998

Prospective	56 48W 8M	37 ± 10	APS (aPL + thrombosis and/or fetal loss) (43 patients with previous arterial/venous thrombosis)	PAPS only	5	14/43 (32.5)	16 (10/6)	Turiel M et al. *Stroke *2005

W – women; M – men; aPL – antiphospholipid antibodies; aCL – anticardiolipin antibodies; LA – lupus anticoagulant; PAPS – primary antiphospholipid syndrome; SLE – systemic lupus erytematosus; Lupus like – Lupus like disease; ITP – chronic idiopathic thrombocytopenic purpura.

### Recurrent venous thrombosis (Table 2)

**Table 2:** Recurrent thrombosis in patients with antiphospholipid antibodies and duration of anticoagulation treatment

**Study design**	**N**	**Mean follow up**	**Results**	**Reference**
Retrospective	70 pt with aPL + arterial/venous thrombosis (1^st ^event)	5.2 years	Recurrence rates (per patient year): ▪ 0.19 for no treatment, 0.32 for aspirin, 0.57 for warfarin INR = 1.9, 0.07 for warfarin 2.0 = INR = 2.9 (p = 0.12) ▪ 0.00 for warfarin INR = 3.0 (p < 0.001)	Rosove MH et al. *Ann Intern Med *1992

Retrospective	19 pt with aPL + venous thrombosis (1^st ^event)	8 years	Probability of survival free from venous thrombosis (Kaplan Meyer method): ▪ 100% in patients kept on warfarin vs. 22% in patients in whom warfarin was stopped – log rank test p = 0.000007	Derksen R et al. *Ann Rheum Dis *1993

Retrospective	147 pt with aPL + arterial/venous thrombosis (1^st ^event)	7 years	Recurrence rates (events per year): ▪ 0.29 for no treatment ▪ 0.015 for INR > 3, 0 for INR > 3 + aspirin (p < 0.001) Probability of survival free from thrombosis (Kaplan Meyer method): ▪ 90% in patients kept on warfarin INR > 3 + aspirin vs. 30% in patients on no treatment (5 years)	Khamashta M et al. *N Eng J Med *1995

Prospective	211 pt with a 1^st ^recurrent venous thrombosis	4 years	Recurrence rates: ▪ 3/15 pt aCL+ (20%) and 20/90 pt aCL- (22%) allocated 6 months warfarin ▪ 1/19 pt aCL+ (5%) and 2/87 pt aCL- (2%) allocated indefinite warfarin therapy	Schulman S et al. *Am J Med *1998

Prospective	162 pt with venous thrombosis (1^st ^idiopathic event) having completed 3 months of warfarin, allocated to either placebo or further 24 months warfarin	10 months	Total of 8 aPL +/150 pt tested (aPL prevalence 5%) Recurrence rates: ▪ 4/6 aPL pt that completed only 3 months warfarin (placebo group) – HR (95%CI) of 4 when compared to patients without aPL. All patients 162; placebo group 83; warfarin group 79	Kearon C et al. *N Eng J Med *1999

aPL – antiphospholipid antibodies; aCL – anticardiolipin antibodies; CI – confidence interval; HR – hazard ratio; INR – international normalized ratio; pt – patients

#### • Risk of recurrence after stopping anticoagulation therapy

In a retrospective study by Derksen et al, 12 of 19 patients with APS and venous thrombosis had recurrent thromboembolic events (63%), all of which occurred in patients in whom anticoagulation had been stopped (median follow up: 8 years) [14].

Recurrent events occurred more frequently in the first 6 months after stopping anticoagulation therapy [15]. Khamashta et al calculated the recurrence rate in this period to be 1.30 events per year [15]. This recurrence rate is higher than in patients with a first idiopathic deep vein thrombosis after 3 months of treatment (0.27 events per year) [24].

Schulman et al conducted a prospective study in which 412 patients without evidence of malignancy or hereditary thrombophilia were anticoagulated for six months after a first venous thromboembolic event and followed up for 4 years [25]. IgG aCL measured once 6 months after the thrombotic events were positive in 15% of the patients. The risk of recurrence after four years was 29% in patients with aCL and 14% in patients without these antibodies (risk ratio 2.1; 95% CI 1.3–3.3; p = 0.0013). The calculated rate of recurrence was 0.10 per year in patients with aCL and 0.04 per year in patients without aCL. A similar risk of recurrence was reported at 10 years of follow up in unselected patients randomly allocated to either 6 weeks or 6 months of warfarin after a first venous thromboembolic event [26].

Kearon et al reported that aPL positive patients having completed 3 months warfarin for idiopathic venous thromboembolism had a hazard ratio for recurrence of 4 (95% CI, 1.2 – 13) when compared to patients without these antibodies (p = 0.03) [24].

#### • Risk of recurrence on anticoagulation therapy

Long term anticoagulation in patients with venous thromboembolism reduces the risk of recurrence and this was confirmed in a meta – analysis [27,28]. Kearon et al quantified a 95% risk reduction when warfarin therapy was extended over 3 months [24]. One meta – analysis, however, has shown that the risk of recurrent venous thromboembolic events decreased over time reaching stabilization at 9 months after the first event independently of the duration of anticoagulation [29]. In fact, the incremental benefit of prolonging anticoagulation was shown to decrease as the duration of anticoagulation increases [28]. Beyond 6 months the magnitude of risk reduction tends to become slimmer and therefore the benefit of maintaining treatment may depend on the estimated individual risk of recurrence [28].

In APS however, the available studies suggest that long term oral anticoagulation therapy is beneficial after a thromboembolic event. Derksen at al reported a 100% probability of being free of recurrence at 8 years in patients with APS and a venous thromboembolic event on anticoagulation therapy compared to 22% in those in whom anticoagulation had been stopped (p < 0.00001) [14]. Khamashta el al, also in a retrospective study, reported that patients with APS kept on oral anticoagulant treatment (target INR = 3) had a 90% probability of being free of recurrences (arterial or venous) over 5 years [15]. Schulman et al prospectively followed up 211 patients tested for aCL randomly allocated to either 6 months warfarin or indefinite anticoagulation after a first recurrent venous thromboembolic event [25]. In the 6 month treatment group 20 out of 90 (22%) patients without aCL and 3 out of 15 (20%) with aCL had recurrences. In the indefinite treatment group, 2 out of 87 (2%) patients without aCL and 1 out of 19 (5%) with aCL had recurrences, although all three recurrences occurred in patients who had stopped anticoagulation [25].

Taken together, these studies suggest that indefinite long term anticoagulation is warranted in patients with venous thrombosis and persistently positive aPL. However, one should be cautious about interpreting the risk of recurrent thrombosis and specifically the risk of recurrent venous events. Firstly, the available data comes from the few studies conducted (3 retrospective studies and 4 prospective studies). Most only included small numbers of patients, many with an underlying systemic autoimmune disease, and had no control groups. In one study, patients were tested only once for aCL and many had low titre antibodies and therefore did not fulfil the criteria for APS. Arterial and venous events were frequently considered together in the evaluation of recurrence and these studies had very different uncontrolled treatment strategies. This can be confusing and hampers the interpretation and generalization of the results. Moreover in none of these studies were other risks factors for thrombosis in patients with aPL extensively evaluated.

All in all, the existing data is not comprehensive enough to strongly support decisions on the optimal duration of therapy after a first venous thromboembolic event or even whether anticoagulation therapy can be safely discontinued in patients with APS. It is not possible to predict which patients will have recurrent thromboembolic events. Ideally, therapy should be continued as long as there is proven benefit for the patient without incurring a significantly increased risk of bleeding. Both in patients with and without APS on warfarin the risk of major bleeding is 2 to 3% per year [7]. Thus optimal duration of treatment is somewhere between the risk of thrombosis and the risk of bleeding.

On the other hand, as far as intensity of treatment is concerned, Crowther MA et al, in a randomized double blind trial including 117 patients with previous thrombosis (mainly venous) fulfilling criteria for APS, demonstrated that high intensity warfarin therapy (target INR 3.1 – 4.0) was not superior to moderate intensity warfarin therapy (target INR 2.0 – 3.0) [30]. Their results suggested therefore that in patients with APS, moderate intensity warfarin therapy is adequate for secondary prevention of recurrent venous thromboembolism.

**Based on the available data, the American College of Chest Physicians recommends for patients with APS and a venous thromboembolic event, warfarin therapy with a target INR of 2.5 (Grade 1A) for 12 months (Grade 1C+), and suggests that indefinite anticoagulant therapy should be considered (Grade 1C) especially for recurrent events **[16].

### Recurrent arterial thrombosis

Based on the retrospective and prospective studies mentioned above (which mostly involved arterial and venous events together) long term, life long, high intensity warfarin therapy is empirically recommended as secondary prevention for persistently positive aPL patients following an arterial thrombotic event (Table 2) [13,15,22,23]. Clinically, ischemic stroke is by far the most frequent arterial thromboembolic manifestation in APS and the risk of recurrent ischemic stroke is considered to be high [7].

#### • Ischemic Stroke

In unselected patients, ischemic stroke tends to occur at older age and in the presence of other risk factors, such as atrial fibrillation, hypertension, high cholesterol, atherosclerosis, diabetes mellitus and smoking [31]. Patient management is directed at secondary prevention of recurrent events with antithrombotic therapy and control of the vascular risk factors. The current standard of care for patients with ischemic stroke and atrial fibrillation is long term adjusted dose warfarin to target INR 2.0 – 3.0 [16,31]. In patients with thrombotic non-embolic ischemic stroke, however, warfarin therapy with a target INR of 1.4 – 2.8 was not found to be superior to aspirin in the prevention of recurrent ischemic stroke, and, in these patients, current recommendations favour antiplatelet therapy [16,32].

In APS, stroke tends to occur at younger ages. In several case series, stroke in patients with aPL was shown to occur at an average age two decades younger than in the general population [33,34]. Indeed, in a recent systematic review, aPL were confirmed to be an independent risk factor for incidental ischemic stroke, although data on their role in recurrent ischemic stroke was found to be weaker and conflicting [34]. Nonetheless, once ischemic stroke occurs, secondary prevention becomes a major issue.

Stroke management in APS patients is a matter of significant debate and controversy. Older restrospective studies have shown recurrent thrombotic events events to be significantly reduced under warfarin therapy with a target INR > 3.0 with or without low dose aspirin [13,15]. Recently, Ruiz-Irastroza et al, in a retrospective case series of 66 patients with definite APS and previous thrombosis (arterial in 77% of patients), reported a high recurrent rate of thrombosis, 9.1 cases per 100 patients-years (95% CI, 3.3 – 19.6), most ocurring at INR between 2.1 and 2.6 [35].

However, in 2003, Dersken et al prospectively followed up 8 patients with ischemic stroke as a first manifestation of APS treated with low dose aspirin for 8.9 years [36]. Recurrent stroke rate was 3.5 per 100 parients years (95% CI, 0.4 – 12.5), a recurrence rate similar to that expected in aPL negative patients with ischemic stroke.

In 2004, the APASS study (Antiphospholipid Antibodies and Stroke Study) proved to be very controversial in the management of stroke in APS. In this study, a large prospective study within the WARSS study (Warfarin vs Aspirin Recurrent Stroke Study), 1770 aPL positive patients with ischemic non cardioembolic stroke were randomly allocated either to aspirin (325 mg/d) or adjusted dose warfarin (target INR 1.4–2.8) [37]. The presence of aPL among patients with ischemic stroke did not predict subsequent tromboembolic events over a two year follow up (24% recurrent thrombotic events in both aPL and non aPL patents; adjusted RR 0.98; 95%CI 0.80–1.20; p = 0.83). Moreover, the risk of recurrent thromboembolic events in aPL positive patients with ischemic stroke was not modified by the type of treatment (aspirin 325 mg versus warfarin with a median INR of 2) [37]. Interestingly, the small group of 120 patients who tested positive for both aCL and LA had a higher risk of thromboembolic events or death regardless of the type of treatment (RR 1.41; 95%CI 0.99–2.02; adjusted p = 0.06; LA × aCL interaction adjusted p = 0.02) [37]. The authors' main conclusions were: a) LA or aCL testing was not important for prognosis or treatment of unselected patients with recent ischemic stroke; b) in patients with stroke who tested positive for aPL, warfarin therapy with targer INR 1.4 – 2.8 was not associated with fewer recurrent events than aspirin.

Several important flaws in this study hamper the generalization of the results to the APS population [37,38]. The average age of the study group was above 60, which is higher than most APS patients groups. Testing for aPL was done only once and the aPL titres were usually low. As such, the classification criteria for APS were not met. Moreover, the empirically recommended high intensity warfarin treatment (target INR > 3) or even the moderate intensity warfarin treatment (target INR 2–3) was not tested. Their inclusion as treatment arms might have led to different results. As a result of these flaws, this study has been widely discredited by opinion leaders as being unreliable evidence on which to base recommendations for stroke therapy in APS patients.

#### • Non cerebral arterial thrombotic events

Virtually any other arterial vascular territory may be affected in APS. Examples include coronary artery disease, namely acute myocardial infarction, intestinal ischemia and glomerular thrombosis, among others. The optimal treatment of these patients is uncertain and management is based on expert opinion. Currently, long term indefinite warfarin therapy (target INR 2.0 – 3.0) is empirically recommended [7].

Overall, these results are not strong enough to modify the current expert based treatment recommendations for secondary prevention of arterial thromboembolism in APS.

Data from the APASS however suggests that older patients with a first ischemic stroke and a single positive aPL on repeated testing and no other risk factors such as atrial fibrillation or systemic autoimmune diseases can be managed in the same way as the general population with ischemic stroke.

## Alternative therapies to Warfarin

Warfarin's anticoagulant effect is due to the inhibition of vitamin K and inherent reduction of vitamin K dependent clotting factors. It is the most widely used drug for long term anticoagulation therapy. However it is a cumbersome drug to use. Warfarin has a narrow therapeutic range, many other drugs and diet can interfere with its anticoagulant effect in either direction, and its use is associated with a 3% risk of major bleeding per year [7,39]. Therefore, frequent blood monitoring is needed for dose adjustment and control. Bleeding is known to occur even within the therapeutic range and several factors including hypertension, use of aspirin or nonsteroidal anti-inflammatory drugs, gastrointestinal inflammation or ulceration, older age, patient reliability, warfarin interactions, clotting factor mutations (Factors IX and XI) and von Willebrand mutations, can contribute to the risk of bleeding [38]. Moreover, warfarin is teratogenic in the first trimester of gestation [38].

All these issues make the quest for safe and effective alternatives to warfarin urgent. However, there is very little information on warfarin alternatives in APS. Kaul et al retrospectively followed up 54 APS patients on secondary thrombosis prevention with aspirin, clopidogrel and low molecular weight heparin. Over 2 years, recurrence rates were 5.4, 9.1 and 3.1 per 100 patients years respectively [40].

### • Aspirin and antiplatelet agents

The APASS study identified aspirin as an adequate long term treatment after non cardioembolic ischemic stroke in a selected group of older patients with low positive aPL [37]. Dersken et al reported a small cohort of 8 APS patients in whom recurrent ischemic stroke rate was very low on low dose aspirin [36]. In the APLASA study, a randomized placebo controlled trial, asymptomatic persistently aPL positive individuals (aCL IgG/IgM/IgA > 20 U GPL and/or LA positive) did not benefit from low dose aspirin (81 mg) for primary prevention of acute thrombosis [41]. The overall acute thrombosis incidence rate in the 98 asymptomatic persistently positive aPL study subjects was 1.33 per 100 patients-years; 2.75 per 100 patients-years in the aspirin group and 0 per 100 patients years in the placebo group (p = 0.83) [41].

Other antiplatelets agents, such as clopidogrel, ticlopidine and aspirin/dipyridamole, that have been validated in clinical trials for use in secondary prevention of ischemic stroke and/or coronary artery disease in the general population, have not been tested in APS.

### • Heparins, Factor Xa inhibitors and thrombin inhibitors

Long term secondary prevention of venous thrombosis with low molecular weight heparins is currently recommended in oncological patients [16]. In these patients, low molecular weight heparins were associated with significantly fewer recurrent events when compared to warfarin [42]. Data in non-oncological patients is scarce. Russel et al randomized 737 patients to receive 3 months treatment either with tinzaparin or standard warfarin for deep vein thrombosis and showed similar effectiveness between the two treatments [43]. Long term low molecular weight heparin therapy was associated with significantly less overall bleeding (minor and major) events when compared to standard care with warfarin (13% for tinzaparin vs. 19.8% for warfarin; 95% CI; p = 0.011). Mucosal bleeding was significantly less frequent in the tinzaparin group (p = 0.003) [43].

In APS patients long term low molecular weight heparin therapy has been empirically used when warfarin is no longer possible, usually due to comorbidities that markedly increase the risk of hemorrhage. In the absence of prospective data and clinical trials, a few authors have reported their empiric experience with this strategy as case reports or small case series with conflicting results [38,40,44].

Fondaparinux is a new subcutaneous anticoagulant that indirectly inhibits Factor Xa. It has been shown to be safe and effective in the acute treatment of deep vein thrombosis and pulmonary emboli and also in venous thrombosis prevention in patients undergoing hip and knee surgery [45]. Indraparinux, a once weekly subcutaneous Factor Xa inhibitor is still undergoing clinical trials. Neither have been tested in APS patients.

Emerging oral anticoagulants, direct thrombin inhibitors and direct Xa inhibitors, such as dabigatran, rivaroxaban and apixaban, were developed as oral alternatives to warfarin for extended treatment of venous thromboembolism and are currently in clinical trials [46]. The role of dabigatran and rivaroxaban in primary prevention of venous thromboembolism after hip and knee arthroplasty has recently been established [47-50]. Indeed, dabigatran is already licenced for use and recommended by NICE as an option for primary venous thrombosis prevention in adults undergoing elective total hip or knee replacement surgery [51].

### • Other drugs

Recently, statins have been a new approach in drug therapy in APS due to their antithrombotic, anti-inflammatory and pleiotropic effects on vascular endothelium [52]. In fact, it has been shown that some statins can block the aPL induced endothelial cell activation which is thought to be a mechanism of thrombus formation in APS [53].

Hydroxychloroquine is widely used in lupus patients and is thought to have a promising role in APS patients. Hydroxychloroquine is known to have weak anticoagulant properties and its use in SLE patients with APS has been associated with a decreased risk of thrombosis [53]. Moreover, it has immunomodulatory effects that might prove beneficial in APS [53].

Some authors have anedoctally reported the use of rituximab in selected APS patients when standard treatment had failed, some with favorable outcome [54,55].

Unfortunately, much has yet to be done, since none of these drugs have yet been studied in clinical trials with aPL positive patients and effective alternative drugs still need to be developed for the management of APS with thrombosis.

## Unanswered questions

Evidence based medicine is often difficult to apply to individual patients. This is especially true of randomised controlled trials where patients are so highly selected that they may not reflect routine clinical practice. The following scenarios illustrate the difficulties.

### Scenario 1: a 30 year old lady diagnosed with APS following a left calf deep vein thrombosis after surgery with two positive LA tests more than twelve weeks apart

The duration of anticoagulant therapy after a venous thromboembolic event has evolved towards a multifactorial risk assessment and stratification based decision making aimed at individually tailoring the optimal length of treatment [56,57].

Among the diversity of factors considered in this process, the presence, nature and reversibility of a triggering risk factor is a major determinant of the duration of treatment [56,57]. Patients developing deep vein thrombosis after surgery (a major transient risk factor) are known to have a very low estimated risk of recurrence compared to patients with a permanent risk factor or an unprovoked event [58]. Fifty per cent of patients with APS have a triggering risk factor at the time of a first thrombotic event [17]. However, no study has yet specifically addressed the influence of triggering and additional risk factors on the risk of recurrence in these patients. Thus their management relies on expert opinion.

Additionally, some features of APS might prove useful in risk stratification. For instance, LA is more strongly associated with thrombosis than aCL [10]. Whether this difference prevails when only recurrent thrombosis is concerned is not known.

It is also unclear whether patients with APS associated with SLE have a higher risk of recurrent events than patients with APS without any underlying systemic disease. Patients with SLE and LA have a 50% chance of having an arterial or venous event at 20 years of follow-up [59]. However, as documented by Petri and Calvo-Alén et al, SLE itself increases the risk of venous thromboembolic events [60,61]. Twenty-one out of 760 SLE patients with active disease (in the Hopkins Lupus Cohort) had a first venous thromboembolic event during follow up, the incidence rate of venous thrombosis being 1.0 per 100 person-years (95% CI, 0.6 – 1.5) [60]. Fifty-one (9%) of 570 patients with SLE in the LUMINA cohort developed venous thrombosis after the diagnosis of SLE [61]. Disease activity was independently associated with venous thrombosis (HR 1.106, 95% CI 1.008 – 1.1213, p = 0.032) and the venous thromboembolic events tended to occur early, rather than late, after the diagnosis of SLE [61].

Following these observations, Merkel et al demonstrated that patients with Wegener's granulomatosis are at increased risk of venous thromboembolic events. The calculated incidence rate was 7 per 100 person-years (95%CI, 4.0 – 11.4), which is much higher than the incidence in patients with systemic lupus erythematosus [1.0 (95% CI, 0.6–1.5)] and rheumatoid arthritis [0.26 (95% CI, 0.1–0.5)] [60,62]. Additional risk factors for venous thromboembolism, namely aPL, were not evaluated. However, APS can occur in association with primary systemic vasculitis. In a cross sectional study, aPL (aCL and/or LA) were present in 25 (17%) out of 144 patients with primary systemic vasculitis and 9 (6%) of 144 fulfilled criteria for APS mainly due to arterial or venous thrombotic events [63]. Nevertheless, just having Wegener's Granulomatosis itself seems to be a risk factor for venous thrombosis. In Merkel et al's study, the venous thromboembolic events tended to occur during periods of active disease or within 2 months of a disease flare [62].

In the past few years, a consistent and progressive increase in the risk of recurrence has been associated with elevated levels of d-dimers obtained one month after discontinuation of anticoagulant therapy for a first venous thromboembolic event, and this risk was reduced by resumption of anticoagulation [64]. In fact, d-dimer levels may prove to be a useful tool in a risk stratification strategy and it could be interesting to assess their role in the context of APS [64].

Clinical practice point 1: This patient would be on indefinite long term warfarin with a target INR 2.0 – 3.0. Moreover, and if laboratory criteria are based on positive LA, once the patient is on warfarin repeat testing is not possible, and though it can be done in highly specialized centres the result can be difficult to interpret.

### Scenario 2: An 18 year old woman is diagnosed with APS following pulmonary emboli while on the oral contraceptive pill

Oral estrogen use in women is an established risk factor for venous thromboembolism. Combined oral contraceptives are known to increase this risk by about two to six fold in pre-menopausal women and hormone replacement therapy by two to four fold [65]. Moreover, some women who develop deep vein thrombosis while taking combined oral contraceptives may have undetected APS [66].

The diagnosis of APS with a venous thrombosis in the setting of combined oral contraceptive use and positive aPL raises two major issues: the optimal length of anticoagulation therapy and alternative contraception methods.

In the absence of an evidence based risk stratified approach to predict recurrent events, current empiric recommendations favour indefinite long term warfarin therapy in these patients. As previously stated, available data on recurrent venous thromboembolism in APS does not address the optimal duration of anticoagulation and nor does it include extensive evaluation of risk factors other than aPL. In many cohorts, there are patients who for several years do not have a recurrent event following discontinuation of warfarin after a single thrombotic event perhaps related to a well documented risk factor. These are often patients in whom the diagnosis of APS was established in retrospect as aPL were not tested at the time of the event. Therefore, some experts have proposed that some APS patients can empirically be considered to be at low risk of recurrent thrombosis, in whom warfarin discontinuation could be considered: patients with a single non-critical vascular thrombotic event developing in the setting of a second transient risk factor (oral combined contraceptive or hormone relacement therapy, pregnancy or surgery) and who have been stable (without further thrombotic events) for at least two years [38].

Clearly, venous thromboembolism and aPL positivity absolutely contraindicates further oral estrogen use [67,68]. Combined oral contraceptives have deleterious hemostatic procoagulant effects, related to estrogen content, that favour venous thrombosis [67,69]. Oral progestins, such as chlromandinone acetate, levonorgestrel and desogestrel, do not induce such hemostatic changes and the few available data have shown progesterone only contraceptives to be safe when used at contraception doses [67,70,71]. A multicenter WHO international, case-control collaborative study found no significant increase in odds ratio for venous thromboembolism among progesterone only oral and injectable contraceptive users [72]. Likewise, levonorgestrel only poscoital contraception, progesterone releasing IUD's and subdermal progestogen implants are not considered to increase the risk of venous thromboembolism [67,68]. Conard et al have shown that among 204 women with past venous thrombosis and/or hereditary thrombophilia, a progesterone only oral contraceptive (chormandinone acetate) was not associated with increased risk of venous thrombosis [71].

The Royal College of Obstetricians and Gynaecologists advises against the use of oral combined contraceptives in women with previous venous thromboembolism, and considers the use of progesterone only contraception in these women [73]. Indeed, the existing data comes from studies in healthy indiciduals, in unselected patients with venous thromboembolism and high risk patients with thrombophilia (mainly hereditary) and venous thrombosis. No study has specifically addressed APS patients who need to be managed as being at high risk for venous thrombosis.

Clinical practice point 2: This patient would be on indefinite long term warfarin with a target INR 2.0 – 3.0. She would be advised against the use of estrogen containing contraceptives. Safe and effective alternatives such as progesterone only contraceptives and barrier methods might be considered. Given that she is aPL positive, a clinical assessment for other autoimmune disorders including testing for antinuclear antibodies might also be considered.

### Scenario 3: A 40 year old patient with a pulmonary embolism and persistently low positive aPL

The clinical significance of low titre aCL is unclear. In a prospective study where 412 patients were assigned to warfarin treatment for 6 months after a first venous thromboembolic and were tested at 6 months for aCL, 28% (17 out of 60) of patients with low positive titres (5 < Ig G aCL < 35 GPL U) and 37.5% (3 out of 8) of patients with moderate to high titres (Ig G aCL > 35 GPL U) had recurrent thromboembolic events at 4 years [25]. Nevertheless, in another study in which 360 patients with aPL were prospectively followed up for 4 years no association was found between low titre aCL and recurrent thrombosis [22].

In fact, low titre aCL are not classification criteria for definite APS. Research studies, especially clinical trials, are based on strict classification criteria that are highly specific and not sensitive in order to prevent bias and to enable the drawing of unequivocal conclusions. Therefore, they rarely include patients with low titre aCL, and in those studies that do include them the results have been controversial. However, in clinical practice, in these patients with low aCL titre, management decisions are not clear cut but, nevertheless, do need to be considered.

Clinical practice point 3: Despite the low aCL, given the documented pulmonary embolism and persistence of the aPL, expert opinion would support the use of indefinite long term warfarin with a target INR 2.0 – 3.0 in addition to minimising other risk factors.

### Scenario 4: In a patient with APS and a single previous deep vein thrombosis, aPL become persistently negative on follow up

APS is considered an antibody mediated acquired prothrombotic disorder, i.e., some aPL seem to be pivotal in the pathogenesis of thrombosis, though the mechanisms responsible for the emergence of the pathogenic antibodies are not yet understood [2]. There are several types of aPL but only some have been associated with the occurrence of clinical features and are therefore used in clinical practice: IgG and IgM aCL, LA and anti β2 glycoprotein I antibodies [1,2]. IgA aCL are not usually considered pathogenic. Antiphospholipid antibodies are known to fluctuate with time. Discontinuing anticoagulation might be argued in a patient with one venous thromboembolic event whose aPL become persistently negative but there is no prospective evidence to support this view. The absence of the antibodies could indicate disease remission. On the other hand, the aPL measured in clinical practice may simply reflect increased thrombosis risk and not necessarily the actual pathogenic mechanism. At present, there is absolutely no evidence to support management decisions.

Clinical practice point 4: In the setting of recurrent thromboembolic events, this patient would be on indefinite long term warfarin irrespective of the aPL status over time. In this patient with a single venous thromboembolic event, discontinuing warfarin therapy might be discussed individually with the patient, with a careful explanation of a risk assessment and stratification strategy for defining the optimal duration of anticoagulant therapy. In the context of an associated autoimmune disease, especially SLE or Wegener's granulomatosis, or other significant cardiovascular risk factors, there would be a compelling evidence based case for continuing indefinite long term warfarin even if the aPL became negative.

### Scenarios 5: A 60 year old woman with APS on warfarin following multiple pulmonary emboli has upper gastrointestinal bleeding from peptic ulceration

Bleeding is by far the most feared complication of any anticoagulant therapy. Patients on warfarin have a 3% yearly risk of major bleeding, which may occur even within the therapeutic INR range (INR 2.0 – 3.0) [7,21]. Therefore, weighing the benefits of anticoagulation against the risk of bleeding remain a constant concern especially as patients age and acquire other bleeding risks and co-morbid conditions.

On the whole, three situations may indicate anticoagulation treatment to be reconsidered in patients on long term warfarin: i) hemorrhagic complications; ii) recurrent thromboembolic events despite optimal anticoagulant management; iii) unmanageable INR not attributable to lack of adherence to medication or diet or drug interactions.

One rational clinical option might be to stop warfarin and switch to long term low molecular weight heparin while new, safe, effective and less cumbersome anticoagulant drugs are being developed [20,21,74]. Low molecular weight heparin treatment implies daily subcutaneous injections but, unlike unfractioned heparin, does not need regular monitoring or dose adjustments and is associated with a significantly lower risk of heparin induced thrombocytopenia and osteporosis [42]. Nonetheless, regular platelet monitoring is needed and osteoporosis risk assessment and prevention is recommended for good clinical practice, when indefinite long term treatment is being considered.

The studies on long term low molecular weight heparin have addressed mainly oncological patients, in whom they were shown to be more effective than vitamin K antagonists for preventing recurrent venous thromboembolism without increased bleeding [42]. Few data are available concerning non-oncological patients with deep vein thrombosis [42]. In patients with APS long term treatment with low molecular weight heparin has been increasingly suggested as an alternative to warfarin in selected patients. Dentali et al anecdotally reported the use of long term low molecular weight heparin in two patients with APS and recurrent thrombosis despite optimal anticoagulant therapy with successful outcomes [44]. However, no specific consistent data is available and its use in these patients needs prospective evaluation.

Clinical practice point 5: This patient would stop warfarin until the bleeding risk was controlled. The options would then include either re-starting warfarin at a lower target INR of 2.5, risking recurrent thrombosis but with a lower bleeding risk or switching to indefinite long term low molecular weight heparin adjusted to body weight. Platelet counts would be regularly monitored (once a week in the first month and then once a month). Osteoporosis prevention and regular screening would be recommended.

## Conclusion

In the antiphospholipid syndrome, the optimal duration of warfarin therapy for secondary prevention of recurrent venous thrombosis after a first venous thromboembolic event remains controversial. Indefinite, life-long warfarin therapy is currently the recommended standard of care. The best available data to support this recommendation have limitations and several questions remain unanswered. Discontinuing anticoagulation therapy in these patients is presently not evidence based and may only be considered in highly selected patients after careful counselling and risk assessment. Likewise, it is unclear for how long this therapy should be maintained. Indefinite long term anticoagulation is considered beneficial for the patient as long as the risk of bleeding does not outweigh the benefits of treatment. Management decisions therefore merge between the available evidence and expert opinion: evidence based versus eminence based medicine.
